# Stigmatising Attitudes among People Offered Home-Based HIV Testing and Counselling in Blantyre, Malawi: Construction and Analysis of a Stigma Scale

**DOI:** 10.1371/journal.pone.0026814

**Published:** 2011-10-26

**Authors:** Peter MacPherson, Emily L. Webb, Augustine T. Choko, Nicola Desmond, Kondwani Chavula, Sue Napierala Mavedzenge, Simon D. Makombe, Treza Chunda, S. Bertel Squire, Elizabeth L. Corbett

**Affiliations:** 1 Liverpool School of Tropical Medicine, Liverpool, United Kingdom; 2 Malawi-Liverpool Wellcome Trust Clinical Research Programme, Blantyre, Malawi; 3 Department of Infectious Disease Epidemiology, London School of Hygiene and Tropical Medicine, London, United Kingdom; 4 HIV Unit, Ministry of Health, Lilongwe, Malawi; 5 Faculty of Infectious and Tropical Diseases, London School of Hygiene and Tropical Medicine, London, United Kingdom; McGill University AIDS Centre, Canada

## Abstract

**Background:**

HIV/AIDS related stigma is a major barrier to uptake of HIV testing and counselling (HTC). We assessed the extent of stigmatising attitudes expressed by participants offered community-based HTC, and their anticipated stigma from others to assess relationship with HIV test uptake. From these data, we constructed a brief stigma scale for use around the time of HIV testing.

**Methods and Findings:**

Adult members of 60 households in urban Blantyre, Malawi, were selected using population-weighted random cluster sampling and offered HTC with the option to self-test before confirmatory HTC. Prior to HTC a 15-item HIV stigma questionnaire was administered. We used association testing and principal components analysis (PCA) to construct a scale measure of stigma. Of 226 adults invited to participate, 216 (95.6%) completed questionnaires and 198/216 (91.7%) opted to undergo HTC (all self-tested). Stigmatising attitudes were uncommon, but anticipated stigma was common, especially fearing verbal abuse (22%) or being abandoned by their partner (11%). Three questions showed little association or consistency with the remaining 12 stigma questions and were not included in the final scale. For the 12-question final scale, Cronbach's alpha was 0.75. Level of stigma was not associated with previously having tested for HIV (p = 0.318) or agreeing to HTC (p = 0.379), but was associated with expressed worry about being or becoming HIV infected (p = 0.003).

**Conclusions:**

Anticipated stigma prior to HTC was common among both men and women. However, the high uptake of HTC suggests that this did not translate into reluctance to accept community-based testing. We constructed a brief scale to measure stigma at the time of HIV testing that could rapidly identify individuals requiring additional support following diagnosis and monitor the impact of increasing availability of community-based HTC on prevalence of stigma.

## Introduction

In sub-Saharan Africa, where two-thirds of the world's people living with HIV reside [1], uptake of HIV testing and counselling (HTC) has been suboptimal [2]. Survey estimates from 18 high HIV-prevalence countries, 15 of which were in sub-Saharan Africa, show that a median of 34% of women and 17% of men have ever undergone testing for HIV and just 12% of women and 7% of men had tested in the past twelve months [2]. Knowledge of HIV status is the key entry point to comprehensive HIV care programmes [3], and from a public health standpoint, is critical to current strategies to reduce HIV transmission, including increasing timely access to antiretroviral therapy (ART) and HIV prevention services [4], [5].

In Malawi, the adult HIV prevalence was estimated to be 11.9% in 2007 [6] and over 300,000 people had initiated ART in the National Treatment Programme between 2003 and June 2010 [7]. However, similar to other high HIV prevalence countries, uptake of HTC has been sub-optimal with only 21% of adults undergoing HTC in 2009 [2]. Strategies to increase awareness of HIV status and decrease barriers to treatment and prevention are urgently needed.

HIV/AIDS-related stigma is a well-documented barrier towards the uptake of HIV/AIDS related services [8], including HTC [9], [10] and ART [11]. Stigma has been conceptualised as the cultural and social differences between members of societies that reinforce power disparities through prejudice and discrimination [12]. Within the context of the HIV pandemic considerable attention has been given to the importance of stigma in acting as a “roadblock to concentrated action, whether at local community, national or global level” [13].

Research on the consequences and public health implications of stigma for people living in high HIV prevalence countries has focused on two areas [14]: stigmatising attitudes (or negative attitudes towards others who may be HIV infected) [15], [16], [17]; and to a lesser extent, anticipated stigma (how individuals feel they would be perceived by others if they were to be diagnosed HIV-positive) [18], . Reliable quantitative measurement of stigmatising attitudes and anticipated stigma experienced around the time of HIV testing could give insights into some reasons for low uptake of HTC. With the increasing availability of ART in sub-Saharan Africa, and the changing context of HIV testing, with WHO recommendations promoting supervised self-testing for health workers [20], a simple scale that could be used to measure trends in stigmatising attitudes and anticipated stigma over time could be beneficial in developing strategies to maximise and support uptake of HIV testing and ART. A number of HIV-related stigma scales have previously been developed but have either focused on a limited number of stigma domains, or have not included participants undergoing HIV testing.

We previously undertook a pilot study to assess the feasibility, acceptability and accuracy of supervised self-testing for HIV using oral mouth swab kits in urban communities in Blantyre, Malawi [21]. Nested within this research, the aims of the current study were to describe patterns of responses to questions regarding stigmatising attitudes and anticipated stigma at the time of direct offer of community-based HIV testing; and to construct a scale measure of stigma using participant responses to provide a simple practical approach that could be used to rapidly identify individuals requiring additional support during and following diagnosis as well as providing a tool to monitor changes in stigma over time, with the anticipated scaling-up of community-based HIV testing in Africa.

## Methods

### Ethics statement

The College of Medicine of Malawi Research Ethic Committee (COMREC) and the London School of Hygiene and Tropical Medicine Ethics Committee granted ethical approval for the study. All participants provided written informed consent to participate.

### Study design, population and procedures

The study was a prevalence study of stigmatising attitudes towards HIV, nested within a cross-sectional community-based random sample survey of feasibility and acceptability of community-based HTC. Community health worker catchment areas (geographical areas defined by the Ministry of Health of Malawi that have a dedicated community health worker responsible for the health of the resident population) in the three high-density residential suburbs of northwest of Blantyre were delineated by circumferential walks, with global positioning system (GPS) mapping of boundaries. Four of 51 catchment areas were randomly selected with probability proportional to size. Satellite maps were used to randomly select 15 dwellings from each of these four catchment areas. Selected dwellings were visited to identify all households (defined as sharing meals). A single household-per-dwelling was then randomly selected, and all adults aged 16 years and over residing within the household were invited to participate in the study.

Individuals who agreed to participate underwent a baseline interviewer-administered questionnaire that collected information on demographic characteristics and experience of previous HIV testing. A stigma questionnaire comprising of 15 questions was undertaken that asked about attitudes towards people living with HIV (“stigmatising attitudes”) and how they felt they would be perceived if they were to be diagnosed with HIV (“anticipated stigma”). Each question was answered on a three-item Likert-type scale.

The stigma questionnaire was based upon the UNAIDS People Living with HIV Stigma Index [22] and stigma questionnaires previously used in studies in Botswana [17], with adaptation to suit locality. We deliberately included questions that addressed areas not previously included in anti-stigma campaigns in Malawi and more broadly in the southern African region as we felt participants may have been less likely to under-report stigmatising attitudes due to a lack of previous sensitisation, or because of desire to give socially acceptable responses to interviewers. Questions were translated into Chichewa and back-translated to English to check consistency.

Following completion of questionnaires, participants were offered three HTC options: self-testing plus confirmatory standard HTC (parallel testing with two rapid finger-prick blood tests), standard HTC alone, or no testing. Pre- and result-based post-test counselling was provided to all participants accepting HTC. Oral self-testing was conducted using OraQuick ADVANCE HIV I/II (OraSure Technologies, Bethlehem, PA, USA) followed by confirmatory finger-prick testing using Determine (Abbott Laboratories, Tokyo, Japan) and Unigold (Trinity Biotech plc, Bray, Ireland), with a third test (SD Bioline HIV I/II Standard Diagnostics, Inc. Yongin-si, South Korea) in the event of discordant results. Data describing test performance and acceptability have been published elsewhere [21].

Participants who were found to be HIV positive were given a referral form with instructions to attend their nearest primary health care centre for comprehensive HIV care, including assessment for ART eligibility.

### Statistical methods: construction and analysis of a scale measure of stigma

Demographic characteristics and responses to stigma questions were tabulated. Chi-squared tests were used to examine associations between each question, and gender differences in question responses.

Principal components analysis of the 15 stigma questions was undertaken to examine patterns of dependence, to assess whether any questions did not fit within the construct that was being measured by the other questions, and to identify whether different constructs were being measured by the “stigmatising attitudes” and “anticipated stigma” questions. Cronbach's alpha coefficient and correlations between each item and the scale formed by the remaining questions were calculated to measure the internal consistency of the questions [23]. Based on these analyses, items that showed low correlation (<0.3) with the scale formed by the remaining questions and which had small loadings on the first principal component were iteratively removed from the scale. Separate alpha statistics were calculated for the blocks of questions that asked about “stigmatising attitudes” towards HIV and “anticipated stigma”. We considered a Cronbach's alpha of greater than 0.7 to represent a reasonable level of internal consistency in this preliminary study [24].

Variables were coded in an ordinal fashion from 0 to 2, with 0 representing the lowest degree of stigma exhibited in a response, and 2 the highest. A stigma score was calculated by taking the sum of the scores for each of the items included in the final scale. After attempting a number of transformations to normalise the data, we recoded the final stigma scale into four approximately equally sized groups corresponding to none, low, medium and high levels of stigma. We used logistic regression to assess the impact of stigma on uptake of HIV self-testing and on worry about being diagnosed with HIV. We used ordered logistic regression to assess the following potential predictors of stigma: gender, age, marital status, education, poverty (as measured by problems getting food in the last month), previous HIV testing and personal knowledge of someone who has died of HIV. Multivariable regression was used to adjust for potential confounders.

Statistical analysis was undertaken using STATA 11.1 (College Station, TX, USA).

## Results

### Uptake of HIV testing among participants

Between March and July 2010, 226 household members were randomly selected and invited to participate in the study; 216 (95.6%) consented to take part and completed questionnaires. Of these 198/216 (91.7%) opted to undergo supervised oral self-testing followed by standard HTC. Baseline characteristics of study participants are shown in Table 1. Levels of education were low in the study population, and reported difficulties in obtaining food were common. Of the 216 participants, 137 (63.4%) had previously tested for HIV, with 47 (21.8%) having tested in the past twelve months.

**Table 1:** Participant characteristics.

		N (%)
Gender	Male	106 (49%)
	Female	110 (51%)
Age	Median (interquartile range)	26.5 (22–32)
Marital status	Married/living with partner	128 (59%)
	Never married	66 (31%)
	Divorced/widowed	22 (10%)
Education (highest)*	None/primary not completed	17 (8%)
	Primary	72 (33%)
	Secondary	111 (51%)
	Higher	16 (7%)
Problems getting food in last month	Never	161 (75%)
	Sometimes	52 (24%)
	Often	3 (1%)
Previously tested for HIV	No	79 (37%)
	Yes	137 (63%)
Personally knows someone who is sick with or has died of AIDS	No	45 (21%)
	Yes	171 (79%)
Chose to have a HIV test in study	No	18 (8%)
	Yes	198 (92%)
Worried about being HIV-positive±	No	170 (79%)
	Yes	42 (19%)

*17 missing values.

±4 missing values.

### Prevalence of stigmatising attitudes and anticipated stigma

Responses to the 15 stigma questions are given in Table 2 (questions 1 to 8 ask after “stigmatising attitudes” and questions 9 to 15 ask after “anticipated stigma” should they be diagnosed HIV-positive). Of the 216 participants, 193 (89%) indicated a stigmatizing attitude or anticipated stigma in response to at least one question. We noted a higher degree of agreement with questions asking after anticipated stigma than for questions on stigmatising attitudes. Nearly one-quarter (47/216, 22% - question 11) feared verbal abuse should they be diagnosed HIV-positive and 14% (29/216 – question 13) thought they would be sidelined by friends. The exception among the stigmatising attitudes questions was question 6, where a high proportion (62.0%) of participants totally agreed that pregnant women should be prevented from having babies.

**Table 2:** Responses to stigmatising and anticipated stigma questions by gender.

	Male		Female		Total		P-value^2^
	N	%	N	%	N	%	
*Q1) Would you buy fresh vegetables from a shopkeeper or vendor if you knew that this person had HIV?*							
Yes	95	89.6	93	84.5	188	87.0	0.326
Don't know	2	1.9	1	0.9	3	1.4	
No	9	8.5	16	14.5	25	11.6	
*Q2) If a teacher has HIV but is not sick, should he/she be allowed to continue teaching?*							
Yes	99	93.4	101	91.8	200	92.6	0.658
Don't know	0	0.0	0	0.0	0	0.0	
No	7	6.6	9	8.2	16	7.4	
*Q3) Would you fear getting HIV from hugging a person with HIV or AIDS?*							
No	100	94.3	100	90.9	200	92.6	0.336
Don't know	0	0.0	0	0.0	0	0.0	
Yes	6	5.7	10	9.1	16	7.4	
*Q4) Would you fear getting HIV from caring for a person with HIV or AIDS?*							
Yes	88	83	93	84.5	181	83.8	0.168
Don't know	0	0.0	3	2.7	3	1.4	
No	18	17.0	14	12.7	32	14.8	
*Q5) A primary school pupil with HIV should not be allowed to continue going to school?*							
No	100	94.3	104	94.5	204	94.4	0.947
Don't know	0	0.0	0	0.0	0	0.0	
Yes	6	5.7	6	5.5	12	5.6	
*Q6) Women with HIV should be prevented from having children*							
Don't agree	25	23.6	38	34.5	63	29.2	0.187
Somewhat agree/don't know	11	10.4	8	7.3	19	8.8	
Totally agree	70	66.0	64	58.2	134	62.0	
*Q7) People with HIV are immoral*							
Don't agree	89	84.0	96	87.3	185	85.6	0.160
Somewhat agree/don't know	9	8.5	3	2.7	12	5.6	
Totally agree	8	7.5	11	10.0	19	8.8	
*Q8) People should not share a meal with a person with HIV*							
Don't agree	103	97.2	104	94.5	207	95.8	0.544
Somewhat agree/don't know	1	0.9	1	0.9	2	0.9	
Totally agree	2	1.9	5	4.5	7	3.2	
*Q9) From what you have seen in your community, if you were HIV positive and people found out, do you think that your wife/husband/partner would leave you?*							
No	65	61.3	69	62.7	134	62.0	0.948
Don't know	29	27.4	30	27.3	59	27.3	
Yes	12	11.3	11	10.0	23	10.6	
*Q10) From what you have seen in your community, if you were HIV positive and people found out, do you think that you would be abandoned or not cared for by family members?*							
No	100	94.3	100	90.9	200	92.6	0.382
Don't know	2	1.9	6	5.5	8	3.7	
Yes	4	3.8	4	3.6	8	3.7	
*Q11) From what you have seen in your community, if you were HIV positive and people found out, do you think that you would be verbally abused?*							
No	81	76.4	81	73.6	162	75.0	0.740
Don't know	4	3.8	3	2.7	7	3.2	
Yes	21	19.8	26	23.6	47	21.8	
*Q12) From what you have seen in your community, if you were HIV positive and people found out, do you think that you would be fired from work or lose your job?*							
No	76	71.7	70	63.6	146	67.6	0.351
Don't know	21	19.8	31	28.2	52	24.1	
Yes	9	8.5	9	8.2	18	8.3	
*Q13) From what you have seen in your community, and you were HIV positive and people found out, do you think that you would be sidelined by friends?* 1							
No	81	77.1	80	74.1	161	75.6	0.823
Don't know	10	9.5	13	12.0	23	10.8	
Yes	14	13.3	15	13.9	29	13.6	
*Q14) If a married person goes for HIV testing, he/she must be unfaithful*							
No	102	96.2	107	97.3	209	96.8	0.325
Don't know	2	1.9	0	0.0	2	0.9	
Yes	2	1.9	3	2.7	5	2.3	
*Q15) From what you have seen in your community, do you think that you would want others to know if a family member became sick with HIV?*							
Yes	48	45.3	29	26.4	77	35.6	0.005
Don't know	9	8.5	6	5.5	15	6.9	
No	49	46.2	75	68.2	124	57.4	

1 3 missing values;

2 Test for different distribution of responses by gender.

Similar response patterns of responses to questions were seen between men and women were found. Of interest, a high proportion of both men (66.0%) and women (58.2%) felt that HIV-positive women should be prevented from having babies (question 6; p = 0.187). Additionally, a similar proportion of men (11.3%) and women (10.0%) feared that their partner would leave them should they be diagnosed HIV-positive (question 9; p = 0.948). Women (26%) were less likely than men (45%) to want others to know if someone in the family was ill with HIV (question 15; p = 0.005).

### Association analysis and principal components analysis

Pairwise associations between responses to the 15 stigma questions were calculated and showed that response to question 6 (“Women with HIV should be prevented from having children”) was not associated with response to any other question. Additionally, question 15 (“Would you want others to know if a family member became sick with HIV”) and question 14 (“If a married person goes for HIV testing and counselling then he/she must be unfaithful”) were associated with only one and two other questions respectively. As anticipated, there appeared to be two main blocks of association corresponding to the two categories of questions (“stigmatising attitudes” and “anticipated stigma”).

Coefficients of the first two principal components obtained in principal component analysis are shown in Table 3. The first principal component consisted of positive coefficients for each question, although the coefficients for questions 6, 14 and 15 are close to zero. The second principal component consisted of negative coefficients for questions 1 to 8 (“stigmatizing attitude” questions) and positive coefficients for question 9 to 15 (anticipated stigma questions). Variation in the third, fourth and fifth principal components were formed largely from questions 6, 14 and 15 (data not shown). Loading plots of principal component 1 against principal component 2 demonstrated the two main blocks of questions (stigmatising attitudes and anticipated stigma), with questions 6, 14 and 15 as outliers (graph not shown).

**Table 3:** Coefficients of first two principal components for 15 questions.

	Principal component 1	Principal component 2
Q1) Would you buy fresh vegetables from a shopkeeper or vendor if you knew that this person had HIV?	0.30	−0.34
Q2) If a teacher has HIV but is not sick, should he/she be allowed to continue teaching?	0.32	−0.28
Q3) Would you fear getting HIV from hugging a person with HIV or AIDS?	0.29	−0.16
Q4) A primary school pupil with HIV should not be allowed to continue going to school?	0.22	−0.13
Q5) A primary school pupil with HIV should not be allowed to continue going to school?	0.26	−0.18
Q6) Women with HIV should be prevented from having children	0.12	−0.07
Q7) People with HIV are immoral	0.36	−0.02
Q8) People should not share a meal with a person with HIV	0.33	−0.34
Q9) From what you have seen in your community, if you were HIV positive and people found out, do you think that your wife/husband/partner would leave you?	0.22	0.32
Q10) From what you have seen in your community, if you were HIV positive and people found out, do you think that you would be abandoned or not cared for by family members?	0.21	0.40
Q11) From what you have seen in your community, if you were HIV positive and people found out, do you think that you would be verbally abused?	0.26	0.35
Q12) From what you have seen in your community, if you were HIV positive and people found out, do you think that you would be fired from work or lose your job?	0.26	0.35
Q13) From what you have seen in your community, if you were HIV positive and people found out, do you think that you would be sidelined by friends?^1^	0.32	0.31
Q14) If a married person goes for HIV testing, he/she must be unfaithful	0.06	0.10
Q15) From what you have seen in your community, do you think that you would want others to know if a family member became sick with HIV?	0.13	0.00

Cronbach's alpha for the combination of all 15 questions was 0.69, and was 0.75 when questions 6, 14 and 15 were removed. We also calculated Cronbach's alpha for two stigma subscales. For the stigmatising attitudes questions Cronbach's alpha was 0.66, but was 0.71 when question 6 was removed. For the anticipated stigma questions Cronbach's alpha was 0.61, but was 0.70 when questions 14 and 15 were removed. On the basis of these findings, for the final stigma scale we excluded questions 6, 14 and 15 and retained the remaining 12 questions. On the final 12-item scale, 111/216 (51.4%) of participants respondents responded with a strong degree of stigma to at least one question.

### Construction of stigma scale and association with HIV testing behaviour

**Figure 1:**
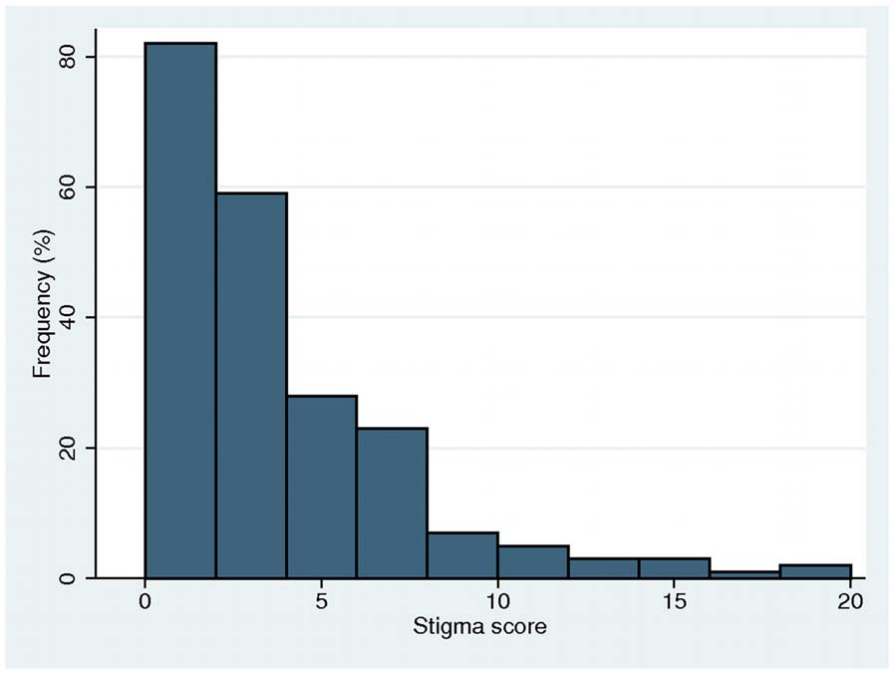
Frequency distribution of stigma scale measurements among 216 participants.

The distribution of stigma scale scores calculated by taking the sum of each of the 12 items included in the final scale is shown in Figure 1. There were three missing values due to 3 non-responders to question 13. Following unsuccessful attempts to transform the highly skewed distribution, we categorised the stigma scale into four approximately equal-sized levels: no stigma = 0; low stigma = 1–2; medium stigma = 3–5; and high stigma = 6–36.

Using the stigma scale as the explanatory variable, in univariate logistic regression (Table 4), we found no association between level of stigma and decision to undergo HIV testing in the study (p = 0.379), but that level of stigma was strongly associated with concern about being HIV positive (p = 0.003). Taking level of stigma as the dependent variable (Table 5), in ordered logistic regression analysis having a higher level of education (p = 0.001) and being younger (p = 0.029) were associated with reduced levels of stigma, but there was no association between having been previously tested for HIV (p = 0.318), personally knowing someone who is sick or has died of AIDS (p = 0.097), gender (p = 0.220), marital status (p = 0.360), problems getting food (p = 0.088) and level of stigma. Concern about being HIV-positive remained associated with stigma, after adjustment for age, education and problems getting food (OR = 1.98, 95% CI: 1.38–2.83, p<0.001). The association between education and level of stigma remained after adjustment for age and problems getting food (OR = 0.59, 95% CI: 0.41–0.85, p = 0.004).

**Table 4:** Univariate associations between level of stigma (explanatory variable) and option to undergo HIV testing and concern about HIV infection (dependent variables).

	Outcome	
Stigma	Chose not to have HIV test	Worried about getting HIV/AIDS
None (N = 59)	4 (6.8%)	5 (8.5%)
Low (N = 67)	5 (7.5%)	14 (20.9%)
Medium (N = 41)	4 (9.3%)	7 (17.1%)
High (N = 42)	5 (11.4%)	15 (35.7%)
Odds ratio (trend) (95% CI)	1.22 (0.79–1.89)	1.63 (1.18–1.25)
P-value (trend)	0.379	0.003

**Table 5:** Univariate associations between level of stigma (outcome) and other factors.

		Stigma					
Characteristic		None	Low	Medium	High	Odds ratio(95% CI)	P-value*
Ever been tested for HIV	Yes	37 (28%)	47 (35%)	26 (19%)	24 (18%)	0.77 (0.47–1.28)	0.318
	No	22 (28%)	20 (25%)	17 (22%)	20 (25%)	1.00	
Personally know someone who is sick or has died of AIDS	Yes	54 (32%)	48 (28%)	33 (20%)	34 (20%)	0.61 (0.35–1.09)	0.097
	No	5 (11%)	19 (43%)	10 (23%)	10 (23%)	1.00	
Gender	Male	30 (28%)	37 (55%)	21 (20%)	17 (16%)	1.35 (0.83–2.20)	0.220
	Female	29 (26%)	30 (27%)	22 (20%)	27 (25%)	1.00	
Age	Mean (SD)	27.9 (9.0)	27.3 (7.6)	28.4 (9.2)	32.2 (13.4)	1.03 (1.00–1.06)	0.029
Marital status	Married/living with partner	31 (24%)	43 (34%)	26 (20%)	27 (21%)	0.71 (0.41–1.22)	0.36
	Never married	22 (34%)	19 (30%)	12 (19%)	11 (17%)	1.00	
	Divorced/widowed	6 (27%)	5 (23%)	5 (23%)	6 (27%)	1.20 (0.52–2.71)	
Education (highest)	None/primary incomplete	1 (6%)	4 (25%)	3 (19%)	8 (50%)	0.55* (0.39–0.77)	0.001
	Primary	15 (21%)	24 (33%)	16 (22%)	17 (24%)		
	Secondary	36 (33%)	36 (33%)	20 (18%)	17 (16%)		
	Higher	7 (44%)	3 (19%)	4 (25%)	2 (13%)		
Problems getting food in last month	Never	50 (32%)	47 (30%)	30 (19%)	31 (20%)	1.00	0.088
	Sometimes/often	9 (16%)	20 (36%)	13 (24%)	13 (24%)	1.60 (0.93–2.76)	

*Trend.

## Discussion

The main findings of this study are that a simple stigma scale was developed with good internal consistency, which had the capacity to separate individuals into those with and without stigmatising attitudes and anticipated stigma. Responses to 3 questions were not included in the final scale due to lack of explanation of variation in the data or lack of internal consistency. Our 12-item final scale constructed through principal components analysis demonstrated good internal consistency when questions asking after both stigmatising attitudes and anticipated stigma were included. The good consistency shown for subscales of stigmatising attitudes and anticipated stigma demonstrate that these more targeted scales could be used within specific situations. However, it will be important to validate both the final scale and subscales in larger and different populations.

Using the final 12-item scale, a higher level of stigma was independently associated with worrying about being or becoming HIV-infected, but was not associated with past HIV test uptake or testing decision, although power to address the latter question was limited by the high HTC acceptance rate of 92%.

Brief stigma scales administered at the time of HIV testing, such as the one constructed, are potentially useful in monitoring changes in stigma over time in sentinel populations (such as ANC attendees [17]), identifying individuals at high risk of severe psychological reactions at the time of testing HIV-positive, or investigating the extent to which stigma is affecting HIV test uptake during community-based interventions.

Among participants in this study, there was a low prevalence of stigmatising attitudes towards others with HIV, with only two of eight items in which more than 15% of participants held strongly stigmatising attitudes. Very similar patterns of stigmatising attitudes have been found in other studies and in other settings. For example, in Tanzania, interviews among randomly sampled community members and purposively selected people living with HIV found that 89% reported they would buy food from a market vendor living with HIV (compared to 87% in our sample) and 95% reporting that teachers with HIV should be allowed to continue work (compared to 93% in our sample) [19]. The low prevalence of anticipated stigma could be a consequence of the widespread community awareness of HIV/AIDS in Malawi [25], and the increasing availability of HTC and ART.

The higher affirmative response to the question “Would you fear getting HIV from caring for a person with HIV or AIDS?” could relate to lack of access to consumables (such as water, soap, gloves and bleach) required for safe and hygienic management of severely ill HIV-infected individuals in impoverished homes. The question “Women with HIV should be prevented from having children” was a newly added question that interestingly had the highest stigmatising response rate (62%). This was not probed further in the current study but may reflect, first, the prominence during post-test counselling given to family planning and the need to avoid unprotected sex to prevent HIV super-infection even within concordant HIV-positive couples and, secondly, widespread awareness about the risk of mother-to-child transmission of HIV resulting from recent scale-up of programs to prevent mother to child transmission of HIV (PMTCT). Previous studies among health workers [26] and the general population [27] that have found that fear of mother-to-child transmission is common and that blame may be apportioned to pregnant women by health workers and members of the general community. However, the right to have children is enshrined under Article 16 of the Universal Declaration of Human Rights [28], and so the high rate of agreement to such a strongly worded statement is concerning and suggests that PLWHA are still subject to discrimination, albeit less crude than in the early days of recognition of the HIV epidemic.

In contrast to the low prevalence of stigmatising attitudes, we found higher levels of anticipated stigma in certain questions, with participants concerned about being verbally abused or being left by their partner if they found to be HIV positive, and not wanting others to know if a family member was sick with HIV. This suggests that, whilst knowledge and awareness of HIV in Malawi has become widespread (with over 99% of adults having heard of HIV and AIDS [25]) leading to increased acceptance of other people living with HIV, the act of undergoing HTC and receiving a positive diagnosis is still associated with considerable fear.

Knowledge of HIV status is the entry point to comprehensive HIV care, including ART. HIV stigma was not associated with the decision to accept community-based HTC in this study; indeed uptake of offer of community-based HTC was very high, a finding which we have previously attributed to the increased convenience and confidentiality of oral self-testing in communities that have a large unmet desire for HTC provided in an accessible and acceptable manner. Stigma was also not associated with having previously been tested for HIV, but was associated with worry about being HIV positive.

These findings firstly suggest that community-based HTC is highly acceptable even among individuals who hold stigmatising attitudes or anticipate stigma. Secondly, there is need to better define the directionality of relationships between stigma, concern about HIV infection and uptake of HIV testing. Some authors have suggested that the lack of access to ART, rather than HIV stigma, is the major factor limiting uptake of HIV testing [29]. We hypothesise that reluctance to test for HIV may result from a combination of factors including: whether or not HTC is directly offered (direct offer being less susceptible to stigma than HTC availability at a facility that requires a proactive decision); other factors such as distance from testing sites affecting ease of access; perceived confidentiality of HIV testing and result-giving procedures; perceived individual control of testing during testing process (i.e. being able to take the test in a home environment and being the first to know the result – as in self-testing); and perceived benefit from testing (including availability of treatment and belief that ART is effective at preventing HIV-related illness and death).

There were a number of limitations of the current study. The initial choice of questions was deliberately kept brief (only 15 questions asked in all), although guided by reported utility in other settings [17]. The high uptake of HTC limited the study power to investigate relationships between stigma and test decision, but could explore association with past testing. Responses may have been affected by social desirability bias (whereby participants tend to give responses that they feel are more socially acceptable), which could have been reduced by self-completed questionnaires or audio-assisted computer interviews. Additionally, there may have been have been a tendency for stigmatising attitudes to be reinforced within households, meaning that attitudes could have been clustered within households. Unfortunately we did not collect data on household so were unable to investigate this possibility further. Nevertheless, it could be argued that in reality, one's family, friends and acquaintances inevitably influence attitudes and as such, these findings give a representation of the true patterns of stigma held within communities, albeit it based on a smaller effective sample size than was actually sampled if household clustering did indeed exist. As we did not ask participants to disclose previous HIV test results to protect confidentiality within the household, we were unable to examine the relation between knowledge of a previous positive HIV test and stigma.

Scales that incorporate questions on anticipated stigma, such as the one developed here, are important to capture all domains of stigma. A central criticism of previous studies that have developed scales to measure HIV stigma is that they have included only one stigma domain within their construct [30], [31], or have questioned only selected groups, such as individuals known to be HIV-positive [32] or pregnant women [19]. A recent systematic review identified 24 published stigma scales that have previously been constructed [18], with seven being based on data from populations in sub-Saharan Africa [30], [33], [34], [35], [36]. A further widely used scale developed from Tanzanian survey data was also identified [31]. However, none of the African scales incorporated responses from HIV-negative individuals asked to indicate how they felt they would be perceived if they themselves were to be diagnosed HIV positive (“anticipated stigma”). A considerable body of evidence exists to suggest that a number of domains of HIV stigma experienced at the individual, community and national level have in the past significantly impaired an individual's ability to seek care for HIV [12]. However, there is also evidence that this is changing with scale-up of HTC and HIV care services [3].

Our data suggest that the availability of community-based HTC could overcome some of these stigma-related barriers to HIV testing. The scale described here could be used to assess the impact of anticipated stigma on newly diagnosed HIV positive individuals' uptake of ART, a major current programmatic limitation within ART programmes in numerous high HIV prevalence countries 37,38,39 Additionally, it is well recognised that receiving a positive HIV diagnosis can be an extremely traumatic and anxiety provoking experience [40], [41] and that individuals with more stigmatising attitudes may experience more severe psychological reactions [41]. The brief stigma scale described here could be used to identify individuals at risk of severe psychological reactions before HIV testing to allow offer of additional interventions and support.

### Conclusion

We describe the construction of a simple scale measure of stigma encompassing both stigmatising attitudes and anticipated stigma that holds promise for a practical approach to quantifying stigma around the time of HIV testing. If generalisable, this scale could be used to monitor the impact of increasing availability of community-based HIV testing on prevalence of stigma over time in high prevalence settings and identify individuals at high risk of adverse experiences during the testing process. Additionally, it could allow investigation of the acceptability and feasibility of different HTC approaches (e.g. community-based versus facility-based) in the same population.
